# Transcriptomics indicate nuclear division and cell adhesion not recapitulated in MCF7 and MCF10A compared to luminal A breast tumours

**DOI:** 10.1038/s41598-022-24511-z

**Published:** 2022-12-03

**Authors:** Jeremy Joon Ho Goh, Corinna Jie Hui Goh, Qian Wei Lim, Songjing Zhang, Cheng-Gee Koh, Keng-Hwee Chiam

**Affiliations:** 1 Bioinformatics Institute, 30 Biopolis Street, Singapore, 138671 Singapore; 2 School of Biological Sciences, Nanyang Technological University, Singapore, 637551 Singapore

**Keywords:** Computational biology and bioinformatics, Data processing, Functional clustering, Gene ontology, Machine learning

## Abstract

Breast cancer (BC) cell lines are useful experimental models to understand cancer biology. Yet, their relevance to modelling cancer remains unclear. To better understand the tumour-modelling efficacy of cell lines, we performed RNA-seq analyses on a combined dataset of 2D and 3D cultures of tumourigenic MCF7 and non-tumourigenic MCF10A. To our knowledge, this was the first RNA-seq dataset comprising of 2D and 3D cultures of MCF7 and MCF10A within the same experiment, which facilitates the elucidation of differences between MCF7 and MCF10A across culture types. We compared the genes and gene sets distinguishing MCF7 from MCF10A against separate RNA-seq analyses of clinical luminal A (LumA) and normal samples from the TCGA-BRCA dataset. Among the 1031 cancer-related genes distinguishing LumA from normal samples, only 5.1% and 15.7% of these genes also distinguished MCF7 from MCF10A in 2D and 3D cultures respectively, suggesting that different genes drive cancer-related differences in cell lines compared to clinical BC. Unlike LumA tumours which showed increased nuclear division-related gene expression compared to normal tissue, nuclear division-related gene expression in MCF7 was similar to MCF10A. Moreover, although LumA tumours had similar cell adhesion-related gene expression compared to normal tissues, MCF7 showed reduced cell adhesion-related gene expression compared to MCF10A. These findings suggest that MCF7 and MCF10A cell lines were limited in their ability to model cancer-related processes in clinical LumA tumours.

## Introduction

Breast cancer (BC) is the most common cancer diagnosed in females, representing 11.7% of global cancer diagnoses with over 2.2 million new BC diagnoses and 680,000 BC-related deaths in 2020^1,2^. Given the concerning global epidemiology of BC, with a projected doubling in incidence to 4.4 million by 2070^3^, research is needed to better understand and treat BC. However, clinical samples are often challenging to obtain and experimentally manipulate. Cell lines are crucial to facilitating scientific experimentation as a limitless source of biological material^4^. They are key experimental models used in exploring cancer biology^5^ and evaluating drugs^6,7^.

MCF10A and MCF7 are the most frequently used cell line models for normal breast tissue and BC tumours respectively^8,9^. MCF10A is a non-tumourigenic human breast epithelial cell line derived from benign proliferative breast tissue, characterised by a lack of estrogen receptor (ER) expression^10^. MCF7 is a widely used in vitro model established from pleural effusion samples from a patient with metastatic BC^11^. It belongs to the Luminal A (LumA) BC subtype^5,12–14^, an ER-positive subtype with the most optimistic prognoses among the BC subtypes^15^.

### Research gaps

Past studies on the clinical relevance of cell lines suggest mixed conclusions which require resolution. A compilation of high-throughput data across hundreds of cell lines from the Cancer Cell Line Encyclopaedia found that cell lines had transcriptional profiles which correlated better with that of their corresponding primary tumours than other tumours, concluding that cell lines represented primary tumours well^16,17^. Within BC, cell lines were found to have expression patterns which correlated with their respective intrinsic subtypes of BC^5,18,19^. However, other studies suggested poor concordance between transcriptomes of cell lines and primary tumours^17,20,21^, and where cell lines correlated more with unrelated cell lines from different cancers than their corresponding primary tumours^22^. These mixed findings call to question the effectiveness of using MCF7 as a model for BC. For MCF10A, two-dimensional (2D) and three-dimensional (3D) cultures of MCF10A were found to express markers which were uncharacteristic of normal human breast tissue^9^, suggesting that MCF10A may be an inappropriate breast tissue model.

We compiled a summary of reported functional differences between cell lines and primary tumours, derived from differential expression analyses between cell line and tumour transcriptomes (Table 1). These studies suggest that cell lines regulated cellular processes differently from primary tumours. Compared to tumours, cell lines displayed upregulation of cell cycle and metabolism, and showed decreased immune, cell adhesion and tissue organisation processes. Moreover, these studies focused on direct differences between cancer cell lines and primary tumours, instead of considering whether the same cancer-related processes could be observed between tumourigenic and non-tumourigenic cell lines, as between primary tumours and non-cancerous tissues. Cancer-invariant gene expression differences between cell lines and primary tumours may be irrelevant to cancer-modelling and should be omitted in the evaluation of cell lines. Additionally, few studies directly validated 3D culture systems relative to primary tumours. 3D cultures are superior to 2D cultures because they retain elements lost in 2D cultures, such as complex cell polarity and lumen formation^23^. 3D cultures of MCF7 result in spheroidal microtissues with luminal-like morphology and express more breast-specific biomarkers compared to 2D cultures^24,25^. Similarly, 3D cultures of MCF10A cells form spheroids with a hollow lumen^26^, introducing phenotypes that would not have been present in 2D cultures^9^. However, few studies compared gene expression of cell lines in 3D cultures to primary tumours. Given the costs of 3D cultures^27^, there is a need to assess its benefits in modelling BC over the less expensive 2D culture systems so that experimenters can better justify switching from 3D culture systems.

**Table 1:** Brief summary of differentially regulated processes between cell lines and primary tumours.

Processes differentially regulated in cell lines compared to primary tumours	Direction	Scope^a^	References
Cell cycle and proliferation	Upregulated	General tumours, BC	^19,20,29,30^
Nucleotide metabolism and RNA production	Upregulated	General tumours	^29^
Glycolysis and energy metabolism	Upregulated	General tumours, BC	^19,20^
Cell communication	Both directions	General tumours	^29^
Immune processes and inflammation	Downregulated	General tumours, metastatic BC	^18,30^
Cell adhesion	Downregulated	General tumours	^20,29^
Tissue organisation	Downregulated	General tumours	^20^

^a^The scope refers to whether the studies focused on BC data, or looked at cancers in general.

### Aims and overview

In this study, we validated transcriptomic data of MCF7 and MCF10A cell lines in 2D and 3D cultures, augmented with previously published experimental data from all RNA-seq and ChIP-seq sample and signature search (ARCHS4) repository^28^, against clinical transcriptomic data of LumA BC and normal breast samples in The Cancer Genome Atlas (TCGA).

**Figure 1:**
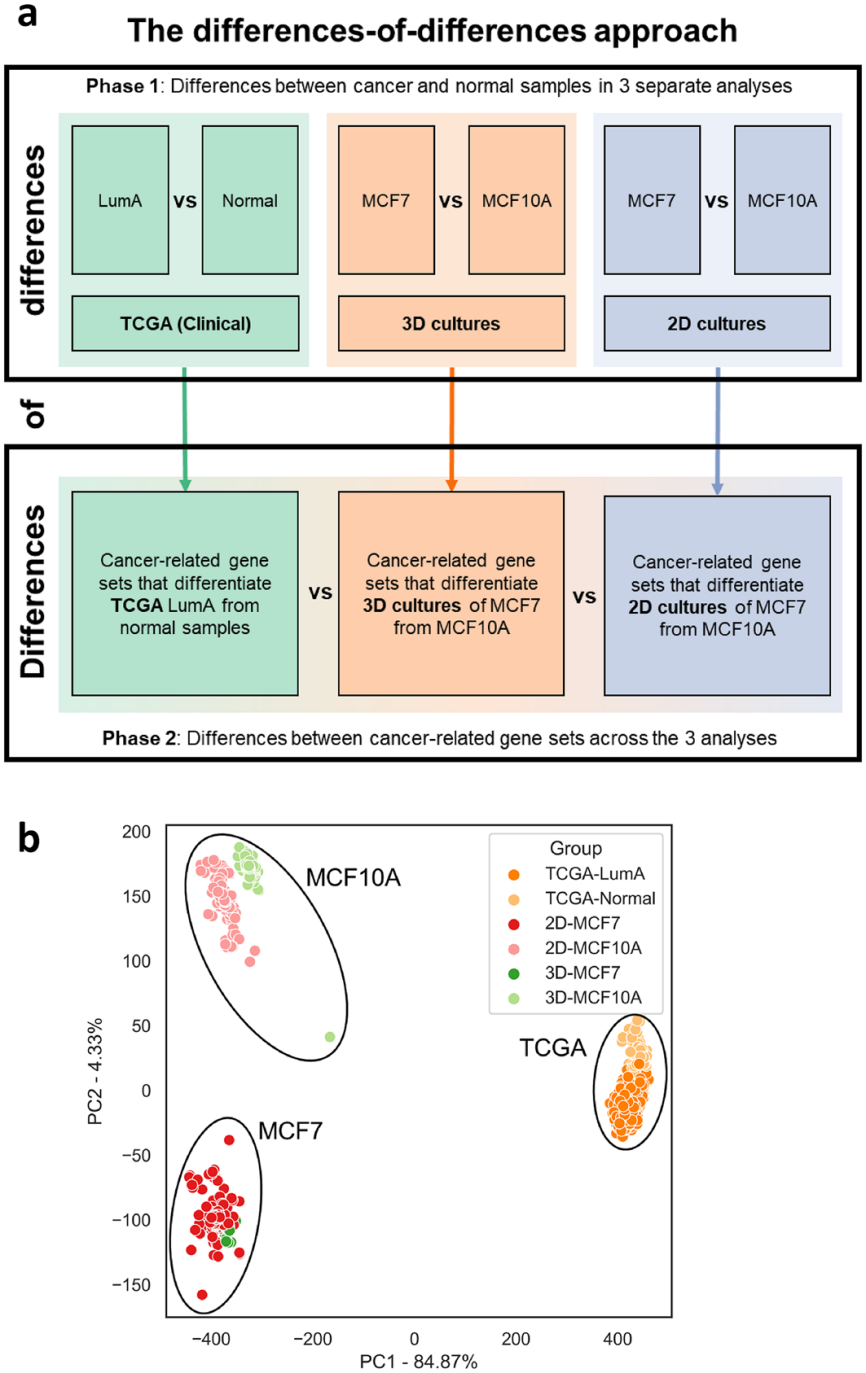
Overview for comparison of cancer progression in cell lines and clinical breast cancer samples. (**a**) Summary of the differences-of-differences approach. (**b**) PCA plot of the first two principal components of the combined dataset of 2D and 3D cultures of MCF7 and MCF10A, and TCGA clinical samples.

We aimed to identify the key cancer-related processes that differ between the cell line analyses (MCF7-vs-MCF10A) and analysis of TCGA clinical samples (LumA-vs-normal), to understand the adequacy of cell lines as models for LumA BC. Unlike past methods which directly considered the differences between tumours and cancer cell lines, our method considers the differences-of-differences between cell lines and clinical BC (Fig. 1). That is, we first identified cancer-related differences via separate analyses of 2D MCF7-vs-MCF10A, 3D MCF7-vs-MCF10A and TCGA LumA-vs-normal, by using a random forest classifier (RFC)-based gene selection approach to select cancer-related genes, and identifying overrepresented biological processes in the selected genes. Thereafter, we compared these overrepresented processes across analyses, to determine how well the comparisons of MCF7-vs-MCF10A were able to represent cancer-related processes in the clinical LumA-vs-normal comparison. We found that analyses of MCF7-vs-MCF10A failed to identify cancer-related changes in mitotic nuclear division, and exaggerated cancer-related dysregulation of cell adhesion compared to clinical LumA-vs-normal. We concluded that MCF7 and MCF10A were limited in their ability to model LumA BC.

## Materials and methods

Data pre-processing and overrepresentation analyses (ORA) were conducted in R version 4.1.2^31^ using RStudio version 2021.9.2.382^32^. Further processing and feature selection were implemented in Python version 3.9.7 using Jupyter Notebook^33^. Where applicable, we specified a random seed of 77 for reproducible analyses. Unless otherwise specified, visualisations were produced with the R package *ggplot2* version 3.3.5^34^, or the Python packages *seaborn* version 0.11.2^35^ and *matplotlib* version 3.4.3^36^.

**Figure 2:**
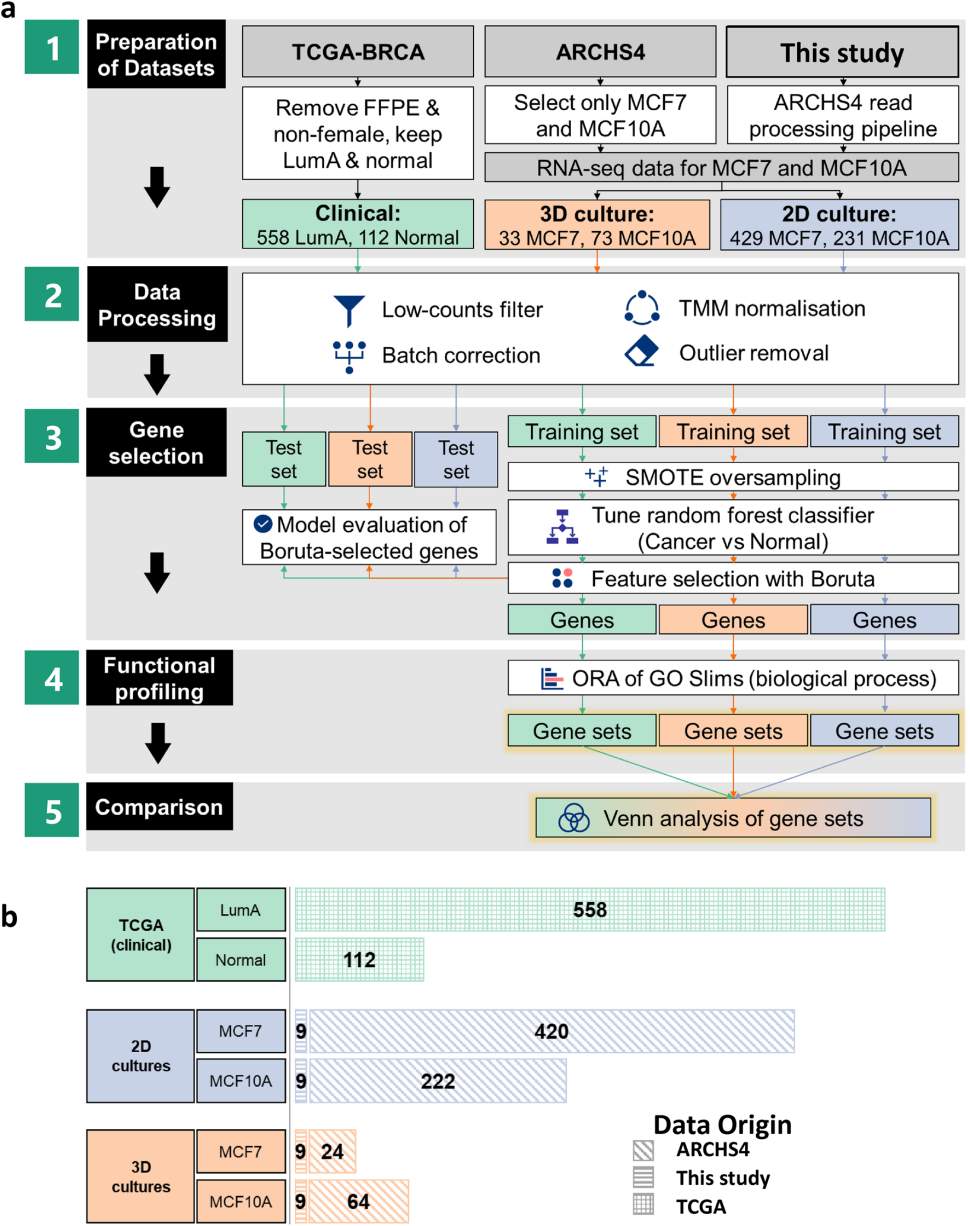
Overall analysis workflow of RNA-seq data. (**a**) Summary of key steps in the analysis workflow. (Refer to “Preparation of datasets” in “Materials and methods” section). *FFPE* Formalin-fixed paraffin-embedded, *GO* Gene Ontology, *TCGA-BRCA* The Cancer Genome Atlas-Breast Invasive Carcinoma dataset, *ORA* Overrepresentation analysis. (**b**) Number of samples by class in the TCGA, 2D culture and 3D culture datasets, respectively. The origins of each dataset are indicated by the textures of the bars.

Our differences-of-differences approach first finds the differences between LumA (or MCF7) and normal (or MCF10A) samples via three separate analyses of the TCGA, 3D culture and 2D culture datasets (Fig. 2a; Steps 1–4), and then compares the significantly overrepresented gene sets across the three analyses (Fig. 2a; Step 5).

### Cell lines and monolayer cultures

The ATCC-originated MCF7 cells (HTB-22) were maintained as monolayer in Dulbecco’s Modified Eagle’s Medium—high glucose (Sigma-Aldrich, USA) supplemented with 10% Fetal Bovine Serum (Gibco, USA), 1% Penicillin/Streptomycin (Gibco, USA). The ATCC-originated MCF10A cells (CRL-10317) were maintained in Dulbecco’s Modified Eagle Medium: Nutrient Mixture F-12 (D-MEM/F-12) (Gibco, USA) supplemented with 5% horse serum (Gibco, USA), 20 ng/ml Epidermal Growth Factor (PeproTech, USA), 0.5 mg/ml hydrocortisone (Sigma-Aldrich, USA), 10 μg/ml insulin (Sigma-Aldrich, USA), 100 nM (−)-Isoproterenol hydrochloride (Sigma-Aldrich, USA) and 1% Penicillin/Streptomycin (Gibco, USA). All cells were passaged or changed with fresh medium every other day, and grown in 37 °C humidified incubator supplemented with 5% CO2.

### Three-dimensional spheroid formation

The spheroids were generated using the 3D ‘on-top’ methods as described previously^37^. Briefly, 8-well chamber slide (Ibidi, Germany) were coated with 50 μl of growth factor-reduced Matrigel (Corning, USA) and left to solidify in the incubator for at least 30 min. MCF7 and MCF10A cells (6500 and 5000 cells respectively) were seeded on top of the solidified Matrigel coating and overlayed with cell medium supplemented with 2% Matrigel (Corning, USA). MCF10A spheroids were cultured in assayed Dulbecco’s Modified Eagle Medium: Nutrient Mixture F-12 (D-MEM/F-12) (Gibco, USA) medium supplemented with 2% horse serum (Gibco, USA), 5 ng/ml Epidermal Growth Factor (PeproTech, USA), 0.5 mg/ml hydrocortisone (Sigma-Aldrich, USA), 10 μg/ml insulin (Sigma-Aldrich, USA), 100 nM (−)-Isoproterenol hydrochloride (Sigma-Aldrich, USA) and 1% Penicillin/Streptomycin (Gibco, USA). Cells were refed with fresh assay medium supplemented with 2% Matrigel (Corning, USA) every two days thereafter. MCF7 spheroids were harvested on day 10, while MCF10A spheroids were harvested on day 16.

### RNA extraction

Total RNA was extracted from MCF7 and MCF10A cells in both 2D and 3D cultures using RNeasy plus kits (Qiagen, Germany) according to manufacturer’s instructions. Briefly, cells grown as monolayer were trypsinized and washed with PBS twice followed by RNA extraction using RNeasy Plus Kit. To collect spheroids, medium was removed from 3D cell culture followed by washing with PBS twice gently. Pre-chilled cell recovery solution (Corning, USA) at the volume of 200 μl were added to 8-well chamber slide (Ibidi, Germany). Matrigel matrix was fully depolymerized, and spheroids were released after 30 min incubation at 4 °C. Suspended spheroids were collected and washed with PBS twice followed by RNA extraction using RNeasy Plus Kit (Qiagen). The integrity and quantity of RNA were determined with NanoDrop 2000 spectrophotometer (Thermo Fisher Scientific, USA) before sending out for RNA sequencing.

### RNA-sequencing

RNA-sequencing was done in two batches with the first batch by BGI Group and the second batch by NovogeneAIT Genomics Singapore Pte Ltd. RNA samples were subjected to quality control (QC) after sample submission to the companies. Preliminary quantification of nucleic acid samples was performed using Qubit (Invitrogen, USA). RNA degradation and contamination were ruled out with agarose gel electrophoresis verification. RNA integrity and quantity were measured on 2100 Bioanalyzer (Agilent Technologies, USA). After sample QC, library construction was performed using NEBNext Ultra II RNA Library Prep Kit (Illumina, USA) according to manufacturer’s protocols. For the first batch, the libraries were run with BGI group’s in-house sequencing platform BGISEQ-500 (20 M single-end reads). For the second batch, libraries were run on NovaSeq PE150 (Illumina, USA) to produce 6 GB of raw data per sample (20 M paired-end reads).

### Preparation of datasets

Raw RNA-seq data comprised of 9 biological replicates per class (3D-MCF7, 3D-MCF10A, 2D-MCF7, 2D-MCF10A). Data from single-end and paired-end sequencing runs were recorded as separate batches. We harmonised the FASTQ file processing by applying the methods in the ARCHS4 documentation^28^. Specifically, *Kallisto* version 0.46.0^38^ with a precalculated human index from the ARCHS4 repository (https://maayanlab.cloud/archs4/, downloaded on 14 March 2022) was used to obtain transcript counts, which were then mapped to gene-level counts using *tximport* version 1.22.0^39^ and the GrCH38.87 human genome annotation^40^.

To improve the generalisability of our findings, we augmented our cell line data with ARCHS4 data. Raw RNA-seq counts from the ARCHS4 repository were downloaded on 21 May 2021^28^, and a subset of MCF7 and MCF10A samples was manually selected based on metadata from the Gene Expression Omnibus (GEO). Only untransformed samples in control conditions were included for further analysis. This dataset was then combined with our RNA-seq data, and subset into 3D culture and 2D culture datasets.

For clinical RNA-seq data, level 3 unnormalised HTSeq mRNA counts data from the TCGA Breast Invasive Carcinoma (TCGA-BRCA) dataset for “primary tumour” and “solid tissue normal” samples were obtained from the Genomic Data Commons (GDC) on 17 January 2022 using *TCGAbiolinks* version 2.22.3^41^. Sample collection and processing protocols are detailed in Ref.^42^. The RNA-seq data had been processed according to GDC’s harmonised pipeline^43^. We discarded formalin-fixed paraffin-embedded samples, as RNA crosslinking due to the fixation process might confound analyses of the higher quality fresh-frozen samples^44^, and discarded non-female samples as male BC are characterised by distinct molecular profiles from female BC^45^. Metadata from^46^ was used for BC subtype assignment. Given that MCF7 is a LumA cell line^12^ and MCF10A is a non-tumourigenic breast cell line^10^, only LumA tumours and normal samples were retained. The number of samples by class in the three datasets are summarized in Fig. 2b.

### Data processing: filtering, normalisation, batch correction, and outlier removal

On each dataset, we performed low-counts filtering, normalised counts using the trimmed mean of M-values (TMM) method^47^, and log_2_-transformed the counts per million (CPM) values using *EdgeR* version 3.36.0^48^, with default settings.

To visualise data structure, we first performed principal component analysis (PCA) on log-CPM data using *scikit-learn* version 1.0.2^49^. We selected the top principal components (PCs) which cumulatively account for at least 80% of total variation as input into the Uniform Manifold Approximation and Projection (UMAP) algorithm^50^. Selecting only the top PCs accounting for majority of variation helps suppress noise^51^, allowing better projection of the relationships between samples. We used the Python package *UMAP* version 0.5.2 with 50 to 200 neighbours, a minimum distance of between 0.05 and 0.3, and random initial embedding positions, to visualise sample relationships. To assess the presence of batch effects and outliers, we generated boxplots by batch along one-dimensional UMAP projections of the PCs (PCA-UMAP) and two-dimensional PCA-UMAP scatterplots. Absolute UMAP values were not provided as they were not intrinsically meaningful. Relative positions of points in UMAP space were more relevant.

One-dimensional PCA-UMAP boxplots of TMM-normalised log-CPM suggested mild batch effects in the TCGA dataset as relative distributions of samples within each class were not homogeneous across plates (Fig. S2a), and substantial batch effects in the 2D and 3D culture datasets because samples tended to cluster by batch without distinct separations between MCF7 and MCF10A samples (Figs. S3a, S4a). Hence, we applied batch correction. Plate IDs (defining separate sequencing runs)^52^ and GEO series IDs (defining separate experiments) were used to specify batch variables for the TCGA and ARCHS4 data respectively. We used the *removeBatchEffect* function in *limma* version 3.50.0^53^ on log-CPM values, specifying the batch variable as described, and LumA (or MCF7) against normal (or MCF10A) samples as the grouping variable.

Consequent to batch correction, distributions across batches highlighted clear differences between LumA (or MCF7) and normal (or MCF10A) samples (Figs. S2b, S3b, S4b), suggesting that most systematic variation due to technical, cancer-irrelevant differences were removed. However, there were 2 LumA outliers in the TCGA dataset (Fig. S2b) and 12 MCF7 outliers (from GSE91395) in the 2D culture dataset (Fig. S3c) which clustered with non-cancer (or MCF10A) samples. These outliers were removed, and the data processing pipeline described above was rerun on the outlier-removed data.

To understand sample relationships across the three datasets, we combined the outlier-removed unnormalised data and kept genes which were present in all three datasets. We then performed low-counts filtering, normalisation and batch correction as described above. We visualised this combined dataset with a PCA plot instead of using PCA-UMAP because the first two PCs had already accounted for over 80% of variation in this dataset.

### Gene selection

To each of the three separate datasets, we applied a random stratified train-test split with a test set size of 20%. Only the training set was used for model fitting.

The number of samples in each class were unequal in all three training datasets (Fig. S1a). This was problematic as class-imbalanced training sets produce classifiers which return biased predictions favouring the majority class^54^. To correct for class imbalance, we applied synthetic minority oversampling technique (SMOTE) on the training sets, implemented in *imbalanced-learn* version 0.9.0^55^, to generate synthetic samples for the minority class based on existing samples^56^. Briefly, SMOTE randomly selected a sample, randomly selected a neighbouring sample out of a specified number (30% of the minority class size) of nearest neighbours in feature space, and generated a random synthetic point between the two samples. This was repeated until the minority class was equivalent in size to the majority class. Two-dimensional PCA-UMAP plots were generated before and after SMOTE to ensure that synthetic datapoints retained data structure.

For each analysis, we tuned a RFC^57^ implemented in *scikit-learn* with 5000 trees using the *GridSearchCV* function, with a pre-specified set of hyperparameters (Supplementary Table 1). In tuning, accuracy was evaluated using a modified tenfold cross-validation, where only the training sets in each fold were oversampled with SMOTE.

To select key genes involved in distinguishing cancer from normal samples, we used *BorutaPy* version 0.1.5, a Python implementation of Boruta^58^, on the SMOTE-oversampled training set and tuned RFC. The implementation entails first generating a set of shadow features by shuffling expression values for each gene. A RFC was fitted on the genes and shadow features to compute Gini importance scores, which quantify how well the given feature classified the samples^59^. Based on whether a gene had a higher importance score than the most important shadow feature, a two-tailed binomial test was performed at each of 1000 successive iterations to determine whether the gene was significantly important (Bonferroni-adjusted *p*-value < 0.0001) in classifying LumA (or MCF7) and normal (or MCF10A) samples. To ascertain that the Boruta-selected genes were cancer-related, we trained the tuned RFC on the Boruta-selected genes from the oversampled training set and used this to obtain an accuracy score in the test set.

### Functional profiling

ORA was performed on the unordered lists of Boruta-selected genes for each of the three analyses using *gprofiler2* version 0.2.1^60^. We used the gene ontology (GO) biological process gene sets from the generic GO slims provided by The Gene Ontology Consortium^61^, downloaded on 11 April 2022. For each analysis, the respective list of genes passing low-counts filtering was used as the background gene list. Gene sets with a Benjamini–Hochberg false discovery rate (FDR) below 0.01 were considered significant.

To determine direction of regulation of the significant gene sets, we first used *limma* version 3.50.0^53^ with *voom* transformation^62^ to compute log_2_-fold changes (LFCs) between LumA (or MCF7) and normal (or MCF10A) samples in each dataset, with batch as a covariate. LFCs were input into a pre-ranked gene set enrichment analysis^63^ implemented in *clusterProfiler* version 4.2.2^64^, using the “DOSE” method with 1000 permutations, without limiting gene set size. We only considered the ORA-significant gene sets. A positive normalised enrichment score meant that the gene set was upregulated, and a negative normalised enrichment score meant that it was downregulated.

### Differences-of-differences: comparison across the three analyses

We compared the results of each analysis by visualising the number of Boruta-selected genes and significant gene sets identified in each analysis using *VennDiagram* version 1.7.1^65^. We selected the top gene sets (ranked by FDR) occurring in the LumA-vs-normal analysis only and the MCF7-vs-MCF10A analyses only for further discussion. Finally, we listed the top genes (ranked by absolute LFC) associated with the selected gene sets, as well as genes which were associated with common gene sets and selected by Boruta in both cell line and TCGA datasets.

## Results

We took a differences-of-differences approach to assess the value of MCF7 and MCF10A cell lines as models for BC (Fig. 1). Contrary to direct comparisons between cell lines and tumours in past studies^19,30^, the key benefit of this approach is the focus on cancer-related processes which are more relevant for tumour-modelling. Comparing cell lines to tumours does not reveal insights about the cancer-modelling ability of cell lines because cancer-related gene expression differences cannot be divorced from general culture-related but cancer-invariant differences. Our comparisons of tumourigenic MCF7 against non-tumourigenic MCF10A would ensure that the identified processes were cancer-related, hence providing a commentary on whether the cell lines model cancer adequately.

### Clinical LumA and normal breast samples had different gene expression profiles from MCF7 and MCF10A

PCA plots of the combined dataset (Fig. 1b) revealed that clinical TCGA samples separated from the cell line samples along the first PC, accounting for 84.87% of variation in the dataset. Hence, majority of variation was driven by differences in gene expression between clinical samples and cell lines, raising doubts about the ability of cell lines to model clinical BC. LumA (or MCF7) and non-cancer (or MCF10A) samples separated along the second PC, suggesting that a minor but substantive amount of variation in the dataset was driven by cancer-related differences. To specifically consider whether these cancer-related differences for cell lines were similar to clinical samples independent of variation driven by broad differences between clinical and cell line samples, we performed separate analyses for TCGA LumA-vs-normal, 3D MCF7-vs-MCF-10A and 2D-MCF7-vs-MCF10A.

### Whether in cell cultures or clinical samples, there were clear cancer-related differences in gene expression

Consistent with the second PC in Fig. 1b, PCA-UMAP projections of the training sets (for three separate analyses) demonstrated that LumA (or MCF7) samples clustered separately from normal (or MCF10A) samples in all three datasets both before and after SMOTE (Fig. S1b). This suggests that majority of variation in the three separate datasets was driven by cancer-related differences in gene expression, and that the oversampled data was able to preserve these cancer-related differences between tumour and normal samples. Hence, genes selected downstream based on these datasets would clearly reflect key cancer-related differences.

### Different genes were driving cancer-related differences between LumA-vs-normal compared to MCF7-vs-MCF10A

We used an RFC-based feature selection approach (i.e., Boruta) to select key cancer-related genes important for classifying cancer from non-cancer samples. Unlike typical differential expression analysis methods which perform independent gene-wise significance tests, RFC-based gene selection methods can demonstrate greater power in large RNA-seq datasets and account for relationships between genes by selecting genes in a multivariate manner^66^. This is especially important given canonical knowledge that cancers involve interactions between genes and pathways^67,68^. RFCs were tuned separately for each analysis, and default settings with 5000 trees produced optimal classifiers. A larger number of trees was chosen to ensure a more robust RFC for gene selection. Cross-validation of the optimal RFCs returned a mean accuracy of 0.998 for the TCGA dataset, and perfect accuracy for both the 2D and 3D culture datasets. This suggested that the fitted RFCs successfully learned the gene expression profiles that distinguished LumA (or MCF7) from normal (or MCF10A) samples.

Unlike other minimal-optimal feature selection algorithms which find the smallest feature subset that maximises classifier performance, Boruta’s all-relevant approach identifies all genes relevant to the classification of cancer from normal samples^58^, allowing a better understanding of the mechanistic underpinnings of cancer. Table 2 lists the number of genes selected by Boruta in each analysis. We validated the classification accuracy of these genes on the test set. Using only the subset of Boruta-selected genes in the tuned RFC fitted on the oversampled training data, all test sets were predicted with 100% accuracy. Given the perfect accuracy in separating cancer (or MCF7) from normal (or MCF10A) samples, we concluded that the chosen genes in each analysis were important cancer-related genes.

**Table 2:** Number of genes (out of those passing the low-counts filter) selected by Boruta.

Analysis	Number of genes passing low-counts filter	Number of Boruta-selected genes
TCGA LumA-vs-normal	24,657	1031
2D MCF7-vs-MCF10A	15,441	450
3D MCF7-vs-MCF10A	15,001	2007

If MCF7 and MCF10A are good models, then they should be distinguished by the same set of cancer-related genes as between LumA BC and normal samples. However, Venn analysis revealed that only 5.1% of genes in the LumA-vs-normal analysis were important in the 2D MCF7-vs-MCF10A analysis (Fig. S5a), and a slightly larger 15.7% of genes in the LumA-vs-normal analysis were important in the 3D MCF7-vs-MCF10A analysis (Fig. S5b). This demonstrates that the genes distinguishing MCF7 from MCF10A were inconsistent with the genes distinguishing clinical LumA from normal samples.

### 2D MCF7-vs-MCF10A had no incremental value in modelling cancer-related processes over 3D MCF7-vs-MCF10A

To understand the biological processes underlying the Boruta-selected genes, we performed ORA within each of the three analyses to identify significant GO slims (FDR < 0.01), and then compared these significant gene sets. Our study focused on gene set-level comparisons because investigations using gene sets are more robust than studies based on genes^69^. We used the generic GO slim, a high-level GO subset intended to give a broad overview of the biological processes involved^61^, because our goal was to summarise the key biological processes differentiating LumA (or MCF7) and normal (or MCF10A) samples. Moreover, using the GO slim reduced redundancy in the gene sets, facilitating comparison across the three analyses.

**Figure 3:**
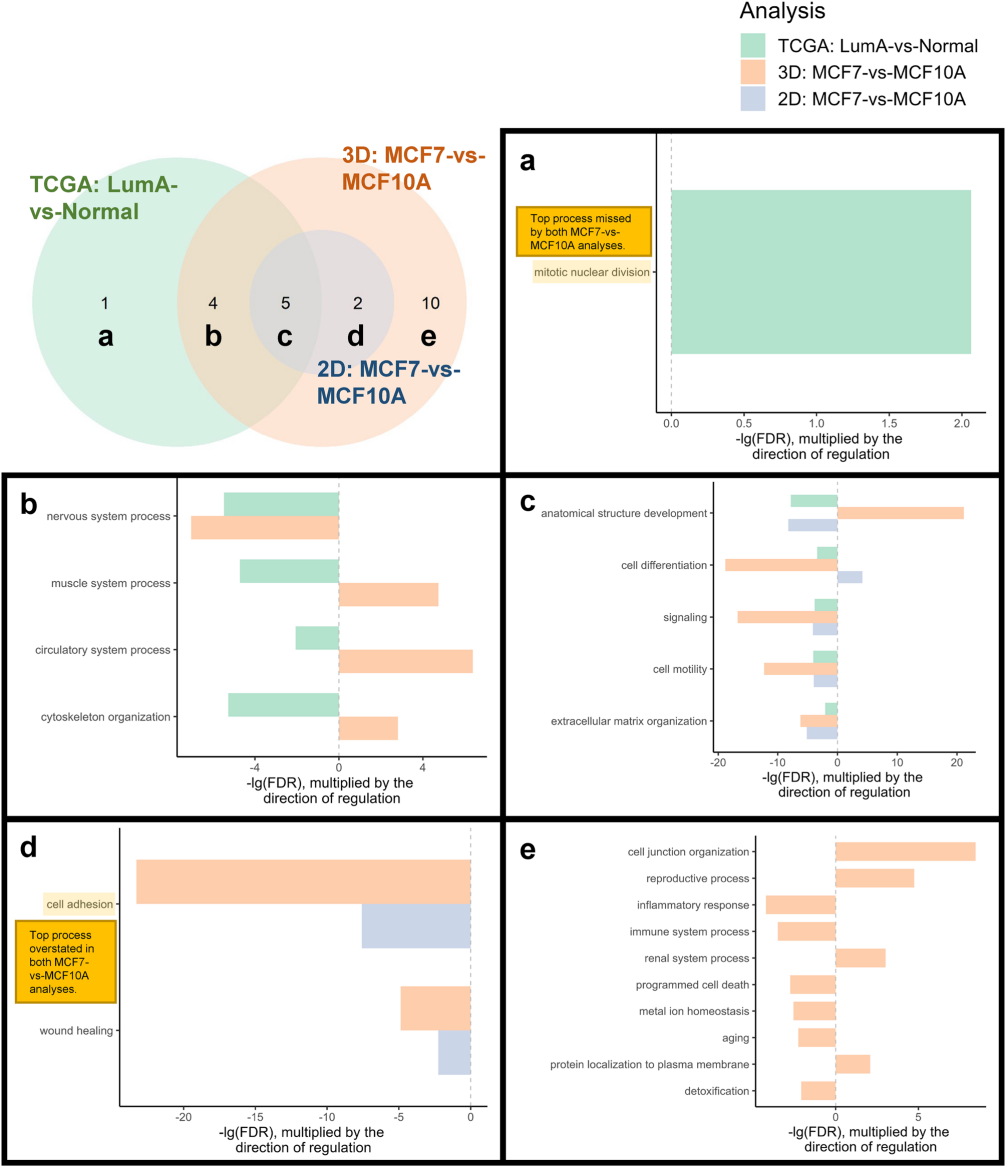
Venn analysis and overrepresented gene sets. (**a**) Gene sets which were specific to the LumA-vs-normal analysis, (**b**) specific to both the LumA-vs-normal and 3D MCF7-vs-MCF10A analyses, (**c**) present in all three analyses, (**d**) specific to both 3D and 2D cultures of MCF7-vs-MCF10A, and (**e**) specific to the 3D MCF7-vs-MCF10A analysis only. For further discussion, we highlighted mitotic nuclear division, the top process missed by both 2D and 3D MCF7-vs-MCF10A analyses, and cell adhesion, the top process overstated in both 2D and 3D MCF7-vs-MCF10A analyses compared to LumA-vs-normal.

Venn analysis (Fig. 3) of significant gene sets indicated that all cancer-related processes in the 2D MCF7-vs-MCF10A analysis were captured in the 3D MCF7-vs-MCF10A analysis. Both the 3D and 2D MCF7-vs-MCF10A analyses were able to successfully identify 5 concordant gene sets with the LumA-vs-normal analysis, including dysregulation of processes related to anatomical structure development and cell differentiation (Fig. 3c). Beyond 2D MCF7-vs-MCF10A, the 3D MCF7-vs-MCF10A analysis was able to successfully identify dysregulation of 4 additional processes present in the LumA-vs-normal analysis (Fig. 3b). Hence, 3D MCF7-vs-MCF10A showed greater consistency in cancer-related processes with clinical LumA-vs-normal, than 2D MCF7-vs-MCF10A with LumA-vs-normal.

### Cell cultures were limited in their ability to model cancer-related processes

We used two criteria to assess the ability of MCF7-vs-MCF10A to model LumA BC at the gene set level. A good model of clinical LumA BC (1) should not miss overrepresentations of cancer-related processes which were found in LumA-vs-normal and (2) should not identify spurious processes which were absent in LumA-vs-normal. Despite similarities in gene sets to the LumA-vs-normal analyses, we found gene sets along these criteria.

#### MCF7-vs-MCF10A did not model cancer-related processes like mitotic nuclear division which were present in clinical LumA-vs-normal

Among the 10 cancer-related gene sets identified in the LumA-vs-normal analysis, the 3D MCF7-vs-MCF10A analysis failed to represent the upregulation of mitotic nuclear division (Fig. 3a). The 2D MCF7-vs-MCF10A analysis failed to represent 6 processes, including cancer-related dysregulation of mitotic nuclear division and nervous system process (Fig. 3a,b). This finding suggests that cell lines, especially in 2D cultures, missed important cancer-related processes. The cancer-related upregulation of mitotic nuclear division was the top process missed by both 2D and 3D cultures.

#### MCF7-vs-MCF10A overstated dysregulation of processes like cell adhesion which were absent in clinical LumA-vs-normal

The 2D MCF7-vs-MCF10A analysis showed dysregulation of 2 additional processes, including cell adhesion, which was not present in the LumA-vs-normal analysis (Fig. 3d). However, the 3D MCF7-vs-MCF10A analysis showed dysregulation of 12 additional processes which were absent in the LumA-vs-normal analysis, including cell adhesion and inflammatory response (Fig. 3d,e). This finding suggests that cell lines, particularly from 3D cultures, tended to overstate the dysregulation of numerous processes. Specifically, the downregulation of cell adhesion was the top process overstated by both 2D and 3D cultures.

**Figure 4:**
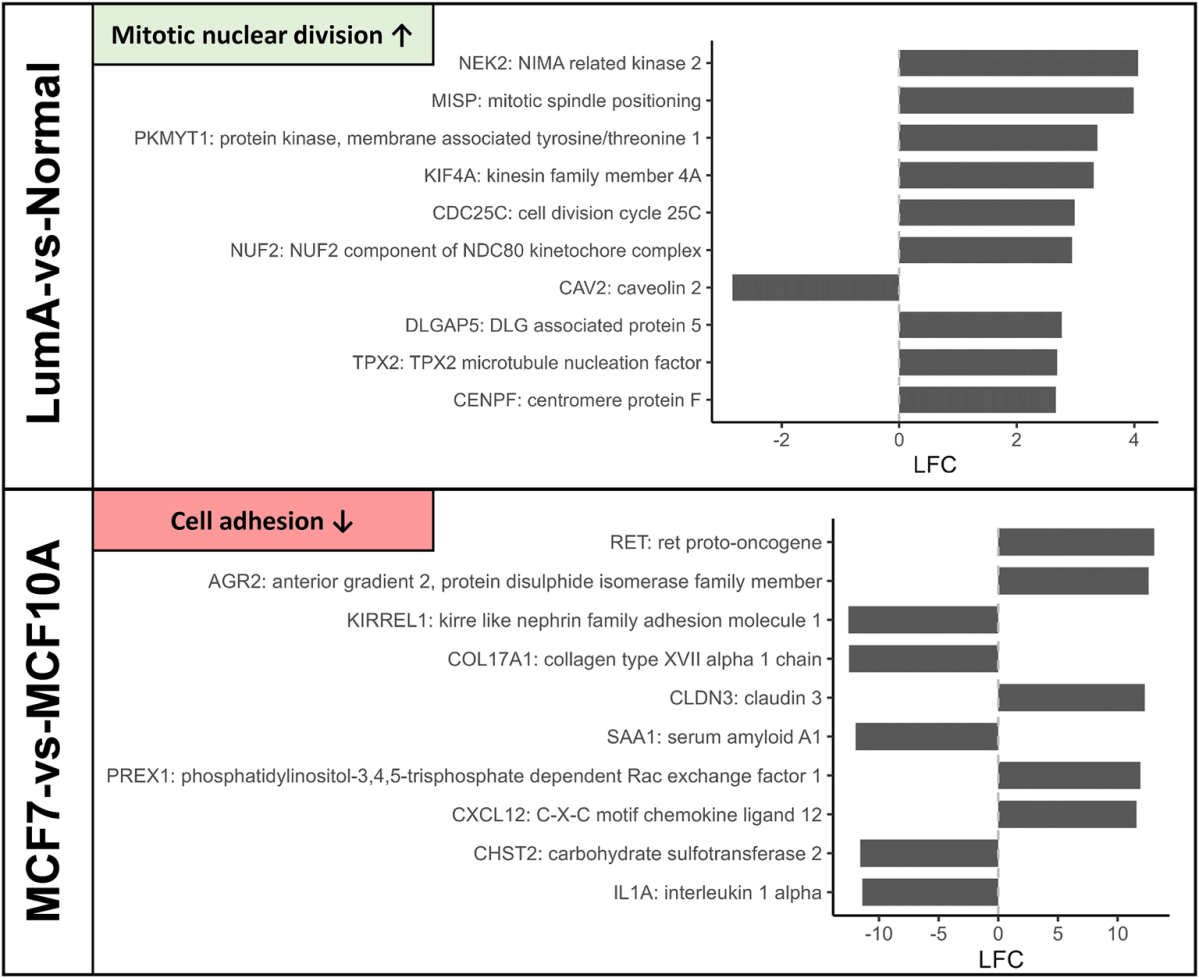
Top Boruta-selected genes which characterise differences in enriched pathways (mitotic nuclear division and cell adhesion) contributing to tumorigenesis in clinical breast tissues and MCF7/MCF10A cell lines respectively. Top panel: Top 10 Boruta-selected genes associated with cancer-related upregulation of mitotic nuclear division in clinical LumA BC compared to normal samples, ranked by absolute LFC (from the TCGA LumA-vs-normal analysis). Bottom panel: Top 10 Boruta-selected genes associated with downregulation of cell adhesion in MCF7 compared to MCF10A, ranked by absolute LFC (from the 3D MCF7-vs-MCF10A analysis).

Finally, we listed the top 10 Boruta-selected genes characterising mitotic nuclear division and cell adhesion (Fig. 4), to explore gene-level differences and propose future experiments such as gene knockdown protocols to improve the modelling ability of MCF7 and MCF10A.

### Common cancer-related genes across cell lines and clinical samples present opportunities for future cell line-based experimental study of LumA BC

**Figure 5:**
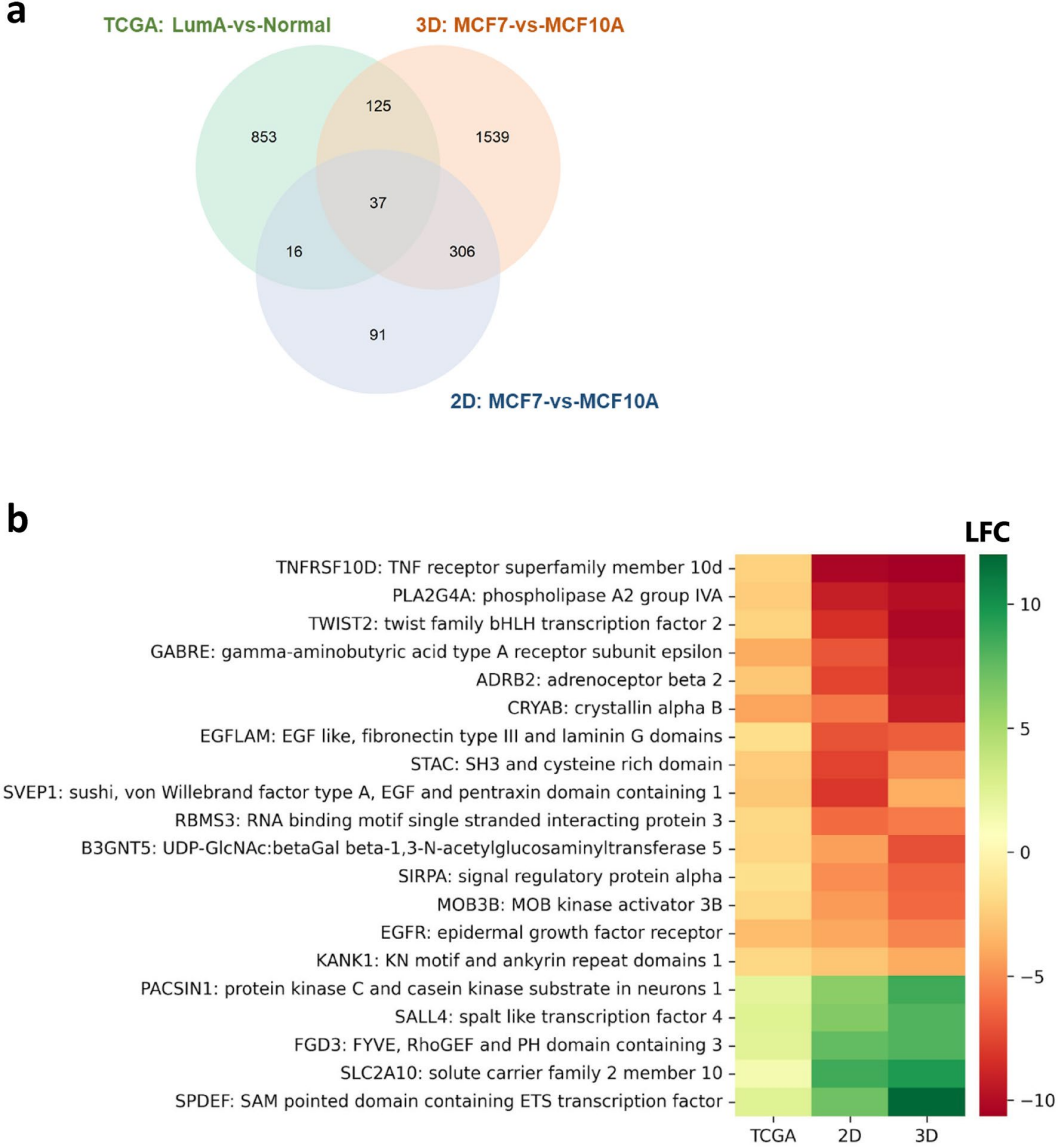
Common cancer-related genes across cell lines and clinical samples. (**a**) Venn diagram showing the number of Boruta-selected genes in each analysis (TCGA: LumA-vs-Normal, 3D: MCF7-vs-MCF10A, 2D: MCF7-vs-MCF10A). 37 genes were selected by Boruta across the three analyses. (**b**) Heatmap of the 20 cancer-related genes which were found to be dysregulated across analyses of 2D and 3D cultures of MCF7-vs-MCF10A cell lines and clinical samples. These genes were sorted by the average LFC across the three analyses. Visualisation was done using the *seaborn* (version 0.11.2) and *matplotlib* (version 3.4.3) Python data visualization libraries.

Beyond differences in gene sets, our analyses presented opportunities for future experimental research. In our comparison between cell lines and clinical samples, we further identified 37 genes (Fig. 5a) which were important in distinguishing cancer from normal samples (i.e., selected by Boruta) in both 2D and 3D cultures of MCF7-vs-MCF10A, and clinical samples. Among these 37 genes, we selected 20 genes (Fig. 5b) which were involved in overrepresentation of biological processes dysregulated across the three analyses (Fig. 3b,c). We sorted these genes by the average of the LFC values across the three analyses.

## Discussion

Our findings suggest that MCF7 and MCF10A were limited in their ability to model LumA BC-related processes, cautioning experimenters about the limited generalisability of these cell line models to real BC. We found low similarity in Boruta-selected genes between cell lines and clinical samples, which suggests that a unique and clinically irrelevant set of genes distinguished MCF7 from MCF10A, compared to the cancer-related genes distinguishing clinical LumA BC from normal breast tissue. Consequently, even when using 3D cultures of MCF7 and MCF10A, researchers may be unable to identify the true set of cancer-relevant genes, and experimental manipulations may end up influencing a set of genes unrelated to clinical BC, leading to wasted time and resources.

### Cancer-related dysregulation of structure development and cell differentiation were effectively modelled in MCF7-vs-MCF10A

However, a further investigation of biological processes identified in each analysis revealed a less pessimistic outlook. Despite the limited similarity in Boruta-selected genes across analyses, these genes were overrepresented for a similar set of processes. For instance, all analyses showed a dysregulation of anatomical structure development. This is consistent with the known loss of structure organisation in BC^70^, characterised by luminal filling of mammary ducts due to unrestricted cell growth^71^, and the findings that developmental pathways are implicated in cancer progression^72,73^. All analyses also showed dysregulation in cell differentiation, consistent with canonical knowledge that cancer cells undergo epithelial-to-mesenchymal transition, a transient state where cells initially dedifferentiate into a mesenchymal phenotype with invasive potential, and subsequently differentiate into various tumour cell types, creating intratumour heterogeneity which facilitates cancer progression and treatment resistance^74–76^. As in the LumA-vs-normal analysis, these patterns of dedifferentiation and differentiation in cancer were correctly represented as dysregulation of cell differentiation in the MCF7-vs-MCF10A analyses.

### 3D cultures of MCF7-vs-MCF10A were superior LumA BC models to 2D cultures, but neither were highly accurate

The 3D MCF7-vs-MCF10A analysis shared more common Boruta-selected genes and enriched gene sets with the LumA-vs-normal analysis compared to the 2D MCF7-vs-MCF10A analysis, suggesting that 2D cultures did not add any value as a model above 3D cultures. The restrictions of monolayer organisation and flat cell morphology in 2D cultures may be responsible for diminished transcriptional patterns^27,77^, resulting in an attenuated set of biological processes differentiating MCF7 from MCF10A. Our findings support the notion that 3D culture systems are superior models to 2D cultures^78^.

Nonetheless, we found that neither the 3D nor 2D analyses accurately represented LumA BC. The 2D MCF7-vs-MCF10A analysis missed half of the cancer-related processes seen in the LumA-vs-normal analysis, suggesting that it was unable to fully capture the complex processes in LumA BC. While the analysis for 3D cultures may have captured most of the processes in LumA-vs-normal, there were 12 additional significant gene sets being highlighted. This suggests that 3D models may lead experimenters to identify many spurious processes which are rare or non-existent in clinical BC, possibly reducing the validity of studies using 3D cultures. Few other studies have investigated whether 3D cultures introduce false positives. Specifically, 3D cultures of MCF10A was found to express markers which were absent in typical breast tissue^9^, further supporting the observation that 3D culture systems introduced changes to cellular processes which differ from breast tissue in vivo. Future work should be done to confirm and understand the elements of 3D cultures that cause cell lines to present spurious processes.

### MCF7-vs-MCF10A comparisons failed to model the cancer-related increase in mitotic nuclear division in LumA-vs-normal

The ability to sustain proliferative signalling is a hallmark of cancer^79^. Therefore, a successful model of cancer should display upregulation in processes related to cell cycle progression. Mitotic nuclear division represents a key step in cell cycle progression^80^. We found that LumA tumours showed greater nuclear division activity compared to normal samples, but MCF7 did not differ in nuclear division compared to MCF10A samples. This suggests that cell lines were unable to accurately model cancer-related cell cycle dysregulation. A possible reason is that both MCF10A and MCF7 were immortalised^10,11^. Immortalisation entails the impairment of cell cycle checkpoints and oncogenic changes^81^, much like cancer cells. Both MCF10A and MCF7 displayed comparable abilities in recovering from cell cycle arrest^82^, suggesting similarities between these immortalised cell lines in cell cycle regulation. Therefore, the cancer-related increase in nuclear division may have been confounded by the immortalisation-related upregulation of nuclear division in MCF10A. Since MCF10A displayed similar upregulation of nuclear division to tumourigenic MCF7, no cancer-related upregulation of mitotic nuclear division was observed in the MCF7-vs-MCF10A comparison.

Our findings are different but not inconsistent with past studies’ findings that cell cycle processes tend to be upregulated in cell lines compared to primary tumours (Table 1). While these studies merely described that cell lines have greater proliferative potential than primary tumours, we found that the immortalisation of cell lines confounded the assessment of cancer-related cell cycle dysregulation, providing a direct commentary on cell line-based cancer-modelling.

MCF7 and MCF10A have been used to understand mechanisms of cell cycle regulation in BC^83,84^. Given that cell cycle pathways are affected by immortalisation, it becomes difficult to determine whether an experimental manipulation affects cancer-related or immortalisation-related cell cycle pathways, weakening the generalisability of these studies to clinical BC. To address the potential confounding influence of immortalisation, experimenters could perform gene knockdowns of mitotic nuclear division-related genes (Fig. 4; top panel) in cell lines to reverse some of the effects of immortalisation prior to experimental manipulation. For instance, *NEK2*, *MISP* and *PKMYT1* are nuclear division-related genes known to be overexpressed in BC^85–87^. These genes should not be similarly overexpressed in non-tumourigenic breast models (like MCF10A). Silencing of these genes may help experimenters create experimental models that better represent non-cancerous tissue.

### MCF7-vs-MCF10A overstated the downregulation of cell adhesion compared to clinical BC

Our findings indicated that MCF7 showed downregulated cell adhesion compared to MCF10A samples, but cell adhesion was not dysregulated in LumA tumours compared to normal samples. This finding was consistent with literature reporting decreased cell adhesion in cancer cell lines compared to tumours (Table 1). Given that the MCF7 cell line was derived from a pleural effusion from a late-stage metastatic BC^88^, the observed downregulation of cell adhesion may have been an artefact of using a cell line of metastatic origin. The loss of cell adhesion-related functions allows detachment from the primary tumour, promoting metastasis^89^. In contrast to MCF7’s metastatic origin, only 1.4% of the LumA samples in the TCGA dataset had been diagnosed as metastatic. Unlike metastatic cells, many primary tumours may retain normal cell adhesion functions, enabling collective cell migration in tumour invasion and facilitating intercellular interactions with other cancer and stromal cells^90,91^. Therefore, cell adhesion processes were intact in the mostly non-metastatic TCGA dataset, but downregulated in the MCF7-vs-MCF10A comparison due to MCF7’s metastatic lineage. Another possible reason is that the standard of care for luminal tumours involves endocrine therapy^92^. The patients in the TCGA were likely treated with anti-estrogen therapy. ER inhibitors like tamoxifen had been shown to restore cell–cell adhesion, reducing tumour invasion^93^. Consequently, this may have recovered cell adhesion in TCGA LumA samples.

To address the differences in cell adhesion due to the metastatic lineage of MCF7 compared to LumA tumours, researchers could use techniques like CRISPR/Cas9-mediated gene activation^94^ or RNA interference-based translational silencing^95^. Candidate cell adhesion-related genes (Fig. 4; bottom panel) for activation include *KIRREL1*, which is known to be overexpressed in BC^96^, and *COL17A1*, whose underexpression is associated with metastatic tumours^97^. Activating these genes may increase resemblance of MCF7’s gene expression profile to that of LumA tumours. Candidate cell adhesion-related genes for suppression include *RET*, which has no known prognostic significance in ER-positive BC^98^, and *AGR2*, whose overexpression is associated with metastasis rather than typical LumA BC, and is known to be inhibited by estrogen inhibitor therapy^99^. The absence of estrogen inhibitor-affected Boruta-selected genes like *AGR2* in the LumA-vs-normal analysis further supports the conjecture that endocrine therapies resolved the dysregulation of cell adhesion-related genes in clinical samples. To further address the differences in cell adhesion due to treatment effects in clinical samples, cell lines modelling post-treatment LumA cancers could be cultured in tamoxifen-treated media, better modelling tumour behaviour under endocrine therapy exposure.

### Opportunities for future cell line-based experimental study of LumA BC

With the compilation of RNA-seq data from the ARCHS4 and TCGA repositories to augment our RNA-seq dataset, we presented a list of putative biomarkers (Fig. 5b) which exhibited concordance in tumour progression for both clinical breast tissues and cell lines.

We could also use this list of genes to pursue more specific questions of interest. For example, to understand the cancer-related morphogenic changes underlying BC, experimenters may be interested in understanding gene expression differences related to extracellular matrix organisation. Among the genes in Fig. 5b, the top shared gene (by average absolute LFC) involved in extracellular matrix organisation was *SLC2A10*. This gene had been demonstrated to be upregulated in clinical BC^100^. *SLC2A10* encodes GLUT10, a facilitative glucose transporter whose role in cancers remains unclear^101,102^. Downregulation of *SLC2A10* is tied to destabilisation of the extracellular matrix via deficiencies in ascorbic acid processing which are cofactors facilitating collagen and elastin production^103^, and impaired cardiovascular morphogenesis via alterations in respiration and TGFβ signalling^104^. However, less is known about the implications of *SLC2A10* overexpression in cancer. The consistent overexpression of *SLC2A10* in cell lines relative to clinical samples suggest that it is possible to further investigate the role of *SLC2A10* overexpression using MCF7 and MCF10A. Future studies could further explore the role of this gene in cancer using experimental cell lines, to better understand its implications on extracellular matrix organisation in cancer cells compared to normal cells.

To further explore cytoskeleton organisation, researchers could analyse *CRYAB*, which was the top cancer-related gene associated with cytoskeleton organisation in the analyses of 2D and 3D cultures of cell lines and clinical samples. *CRYAB* encodes a small heat-shock protein associated with maintaining cytoskeletal integrity under stresses to the cytoskeleton^105^, possibly by reducing the aggregation of F-actin^106^. Studies on *CRYAB* expression in BC were mixed. While *CRYAB* was overexpressed and associated with poorer prognosis in the basal BC subtype and metastasis^107–109^, *CRYAB* was shown to be strongly downregulated in all BC subtypes^110^. Interestingly, separate clusters of BC samples were identified to be varying in *CRYAB* expression, where *CRYAB* tended to be less expressed in ER-positive BC but more expressed in ER-negative BC^111^, suggesting a subtype-specific gene expression pattern in *CRYAB*. While the tumour-suppressive roles of *CRYAB* in LumA BC are less well-established, *CRYAB* has been shown to reduce progression in nasopharyngeal cancers by associating with membrane-bound β-catenin, preventing the release of β-catenin into the cytoplasm, thereby suppressing the oncogenic abilities of β-catenin by preventing it from interacting with complexes to facilitate transcription of genes involved in tumour progression^112^. MCF7 and MCF10A cell lines may be useful models to resolve the mixed findings of *CRYAB* expression in cancers and better understand the mechanisms underlying *CRYAB* in BC. Given how its underexpression in LumA BC was consistently reflected in both cell lines and clinical samples, future work could use MCF7 cell lines to examine the roles of *CRYAB* in LumA BC, such as via gene activation protocols. There is a need for such studies to identify downstream effects of *CRYAB* expression, identify *CRYAB*’s tumour-suppressive role in LumA BC, and better determine whether *CRYAB* is an effector in cytoskeletal organisation or disorganisation.

### Limitations and future work

Our cell line dataset was a collection of independent experiments with varying culture and sequencing protocols. Specifically, in the ARCHS4 data, many MCF7 samples were obtained from different experiments from the MCF10A samples. Despite our attempts to remove cancer-unrelated variation, batch effects may still exist. Nonetheless, our study aimed to look at the broad cancer-related processes distinguishing cell line models from clinical samples and was able to capture the general differences between analyses of clinical samples and cell lines irrespective of remaining batch effects.

Deriving biological meaning from manually curated GO annotations also provides a limited view of tissue-specific functions. It would be worthwhile to seek an alternative method to impute knowledge for uncovering unannotated biological processes for less studied genes.

BCs exhibit high intra-tumour heterogeneity consisting of different cell subpopulations, genetic heterogeneity and mixed morphologies^113–115^. Moreover, cell subpopulations have important implications on treatment efficacy. For instance, studies have found that tumour-associated macrophages are related to worse prognoses^116^ while tumour-infiltrating lymphocytes are associated with good prognoses^117^. These effects of intratumoral heterogeneity cannot be elucidated directly with bulk RNA-seq data. Future work could collate single cell RNA-seq data to compare specific cell subpopulations between tumours and cell lines. This would allow us to answer more specific questions, such as whether cell lines preferentially model certain cellular subpopulations or whether intratumoral heterogeneity in clinical samples introduces complexity that cannot be modelled in cell lines.

Furthermore, our 3D culture dataset was limited in size compared to the other datasets. There are also various sophisticated 3D culture systems which better model tumour-stromal interactions such as co-cultures with other cell types^118^ and vascularised systems^119^, which were not represented in this study. Hence, the list of cancer-related genes and gene sets identified may not generalise well to the full range of 3D culture technology. As 3D culture systems gain popularity, there might be more available data in future which can be used to determine the key cancer-related processes more reliably in 3D MCF7-vs-MCF10A. Future work could amass a larger dataset and further consider the differences between 3D culture techniques, to better assess the modelling capacity of MCF7 and MCF10A.

Finally, our study focused on MCF7 and MCF10A, among the many available BC and non-tumourigenic cell lines. However, our method is scalable and can be easily extended to RNA-seq data mined for other cell lines. Future work can consider expanding implementation to other BC cell lines to obtain a comprehensive idea of the overall representativeness of cell line models to clinical BC data and assist cell line selection in experimental studies by ranking cell lines by similarities in cancer-related processes to relevant breast tissues.

## Supplementary Information

Supplementary Information.

Supplementary Table 2.

Supplementary Table 3.

## Data Availability

RNA-seq data performed in this study for the 2D and 3D cultures of MCF10A and MCF7 were deposited at Gene Expression Omnibus (GEO) repository as GEO Series record GSE208731.

## Abbreviations

2D: Two-dimensional
3D: Three-dimensional
ARCHS4: All RNA-seq and ChIP-seq sample and signature search
BC: Breast cancer
CPM: Counts per million
FDR: False discovery rate
ER: Estrogen receptor
GDC: Genomic data commons
GEO: Gene expression omnibus
GO: Gene Ontology
LFC: Log_2_-fold change
LumA: Luminal A
ORA: Overrepresentation analysis
PC: Principal component
PCA: Principal component analysis
PCA-UMAP: UMAP projection of principal components
RFC: Random forest classifier
SMOTE: Synthetic Minority Oversampling Technique
TCGA: The Cancer Genome Atlas
TCGA-BRCA: The Cancer Genome Atlas Breast Invasive Carcinoma
TMM: Trimmed mean of M-values
UMAP: Uniform Manifold Approximation and Projection
