# Supplementary material for: Transcriptomics indicate nuclear division and cell adhesion not recapitulated in MCF7 and MCF10A compared to luminal A breast tumours
Source: Sci Rep. 2022 Dec 3;12:20902. doi: 10.1038/s41598-022-24511-z (PMC9719475; doi:10.1038/s41598-022-24511-z)
Supplement: Supplementary file 1 — Supplementary Information. [file 41598_2022_24511_MOESM1_ESM.pdf]

Supplementary data

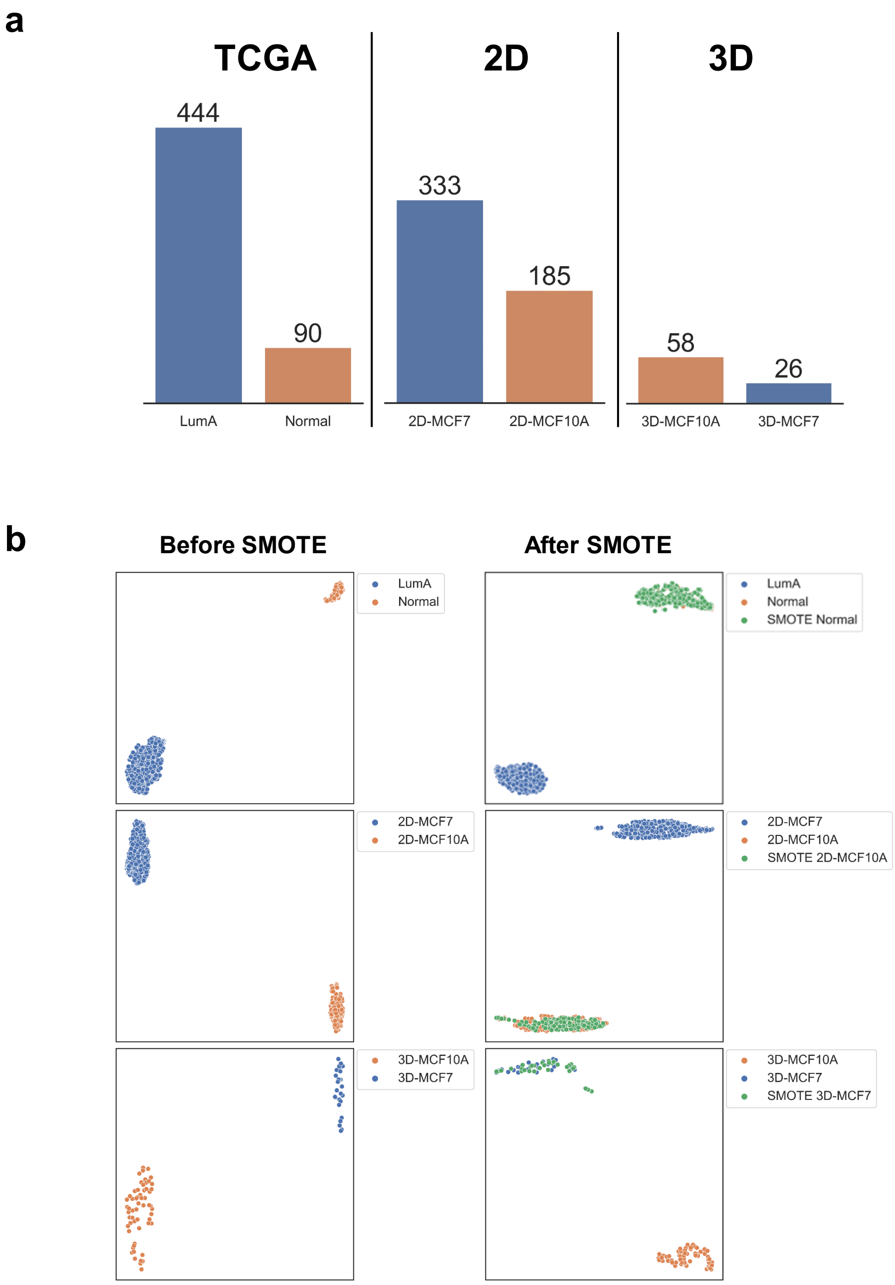

Figure S1. SMOTE oversampling. (a) The number of samples in each class after train-test split in the TCGA (left panel), 2D culture (middle panel), and 3D culture (right panel) training datasets, respectively. (b) Two-dimensional PCA-UMAP plots of the training set before and after SMOTE for TCGA (top panel), 2D culture (middle panel) and 3D culture (bottom panel) datasets, respectively.

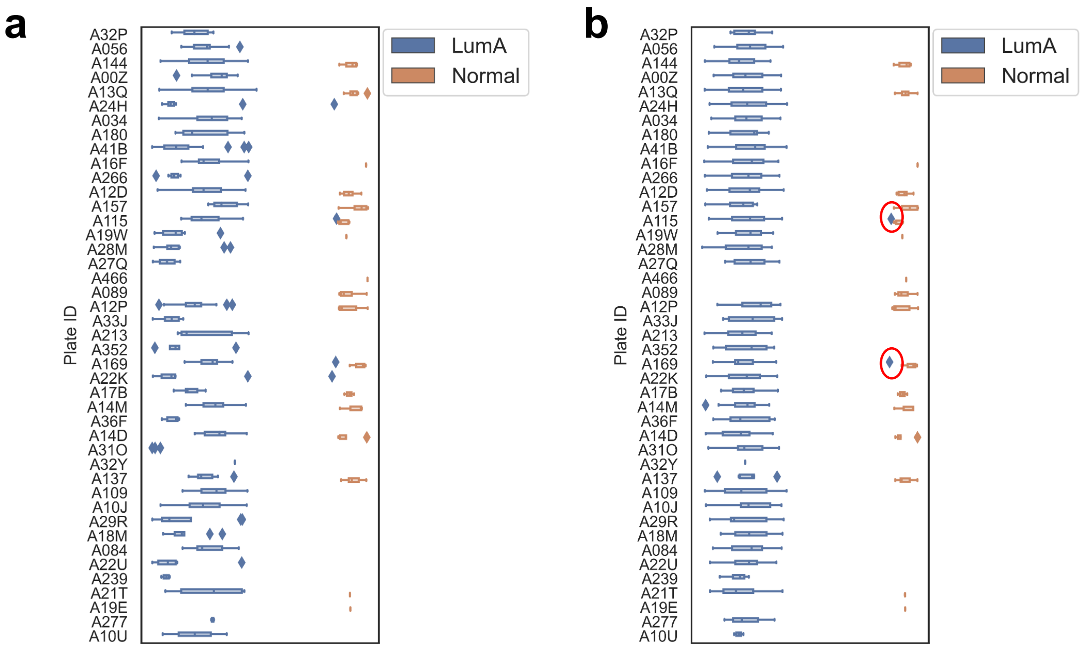

Figure S2. One-dimensional PCA-UMAP projections of TCGA samples as a boxplot (a) before and (b) after batch correction (with outlier samples circled in red).

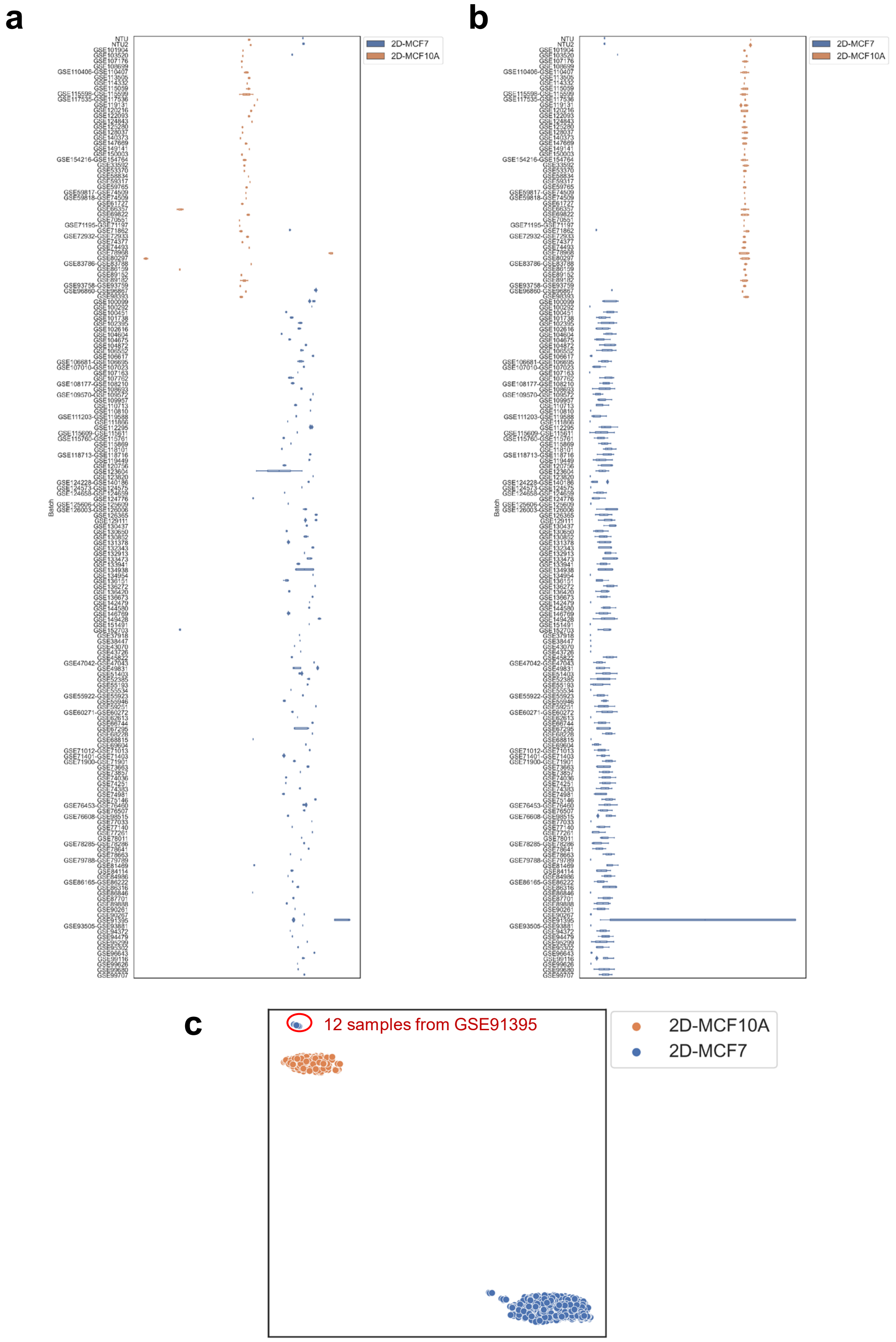

Figure S3. PCA-UMAP projections of 2D culture samples. (a) One-dimensional PCA-UMAP projections of 2D culture samples as boxplots before and (b) after batch correction, and (c) a two-dimensional PCA-UMAP projection of 2D culture samples after batch correction to visualise outliers (circled in red) more clearly.

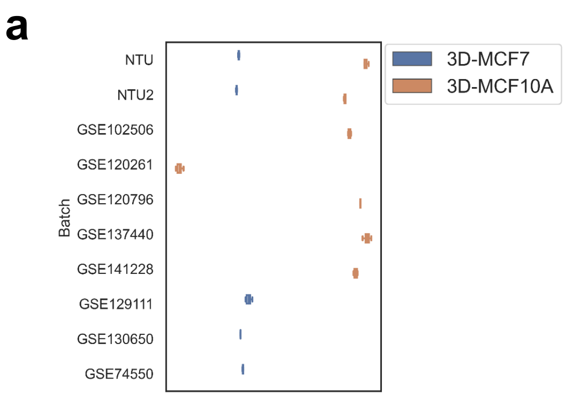

Figure S4. One-dimensional PCA-UMAP projections of 3D culture samples as boxplots (a) before and (b) after batch correction.

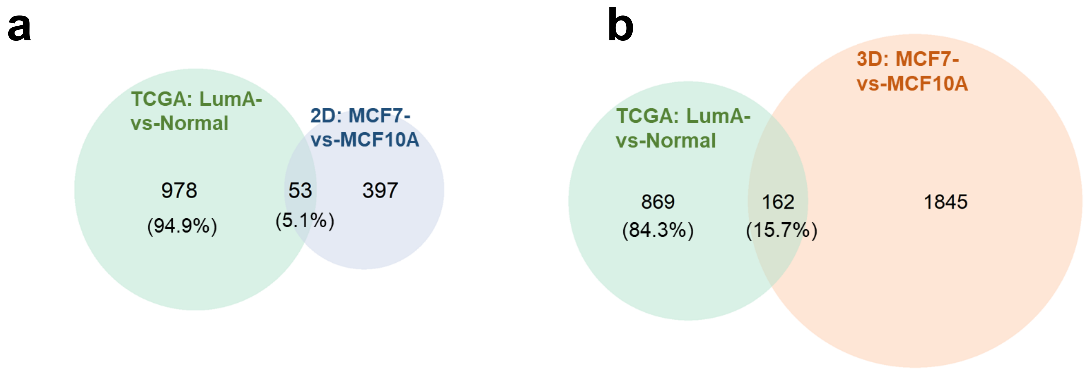

Figure S5. Comparison of the number of shared and unique Boruta-selected genes between the (A) LumA-vs-normal and 2D MCF7-vs-MCF10A analyses, and (B) LumA-vs-normal and 3D MCF7-vs-MCF10A analyses. The percentage of Boruta-selected genes in the LumA-vs-normal analysis shared in the MCF7-vs-MCF10A analyses are listed in parentheses.

Supplementary Table 1: Hyperparameters tested in model tuning

Hyperparameter	Description	Values Tested
max_samples	Maximum percentage of the sample size used in bootstrap sampling for each tree in the random forest. "None" entails using 100% of the sample size for bootstrap sampling.	0.5, 0.75, None [a]
criterion	Criteria used to measure the quality of each split in the decision tree and decide how to split the tree.	Gini [a], Entropy
max_features	Number of features considered when deciding each split. The "auto" setting entails using the square root of the total number of features.	0.005, 0.05, auto [a], 0.1
max_depth	Maximum number of splits allowed in each tree of the random forest. The "None" setting entails allowing trees to grow until splits result in pure nodes.	3, 5, 7, None [a]

[a] Optimal parameter (determined from model tuning) used in the RFCs in all three analyses.

Supplementary Table 2: Sample information of 2D and 3D MCF7/MCF10A datasets in this study and curated GEO datasets used for analysis with outliers removed. The two RNA-seq batches performed in this study are labelled under GEO record GSE208731.

Supplementary Table 3: Sample information of curated TCGA datasets used for analysis with outliers removed.
